# Terahertz Amplification Induced by Electron–Phonon Interactions in Gated Graphene Plasmonic System

**DOI:** 10.34133/research.1023

**Published:** 2025-12-03

**Authors:** Zijian Qiu, Shengpeng Yang, Sunchao Huang, Shaomeng Wang, Ping Zhang, Yubin Gong

**Affiliations:** ^1^ National Key Laboratory of Science and Technology on Vacuum Electronics, School of Electronic Science and Engineering, University of Electronic Science and Technology of China, Chengdu, China.; ^2^ Terahertz Radiation and Application Key Laboratory of Sichuan Province, Chengdu 611731, China.; ^3^ Yangtze Delta Region Institute (Quzhou), University of Electronic Science and Technology of China, Quzhou, China.

## Abstract

Terahertz (THz) amplification presents promising potential for various innovative applications. Here, we achieve an ultra-broadband and high-gain THz amplification from 0.1 to 25 THz with a saturated electric field peaking at 10^7^ V/m by leveraging drift-induced plasmon instability in a single-layer gated graphene system. The plasmon instability arises from the electron concentration modulation due to the nonuniformity of the electron–phonon scattering rate distribution on the Fermi surface. The modulation will introduce increments to compensate for the plasmon loss and even achieve plasmon amplification. Specifically, we derive the nonreciprocal plasmon dispersion using Maxwell’s equations in conjunction with the Boltzmann transport equation, incorporating interactions between electrons and phonons. We find that the amplifying plasmon mode propagates against the electron drift velocity, and the amplification efficiency can be tuned by adjusting either the electron concentration or the electron drift velocity. Furthermore, the simulation shows that when the fundamental mode is amplified to saturation, higher harmonics are generated and amplified, forming a THz frequency comb. These results provide a promising avenue for developing tunable on-chip THz light sources, amplifiers, and frequency comb generators within integrated plasmonic systems.

## Introduction

There is growing interest in the development of miniaturized terahertz (THz) light sources, driven by the increasing demand for applications in sensing and imaging systems, biomedical fields, communications, and spectroscopy [1–3]. Surface plasmons in noble metals have demonstrated important potential for the generation and manipulation of nanoscale light sources, covering a frequency range from infrared to visible light [4–8]. To generate THz light through surface plasmons, researchers are increasingly focusing on 2-dimensional (2D) materials with Fermi energies lower than those of noble metals. 2D materials such as graphene, graphite, WSe_2_, and MoS_2_ have shown promising applications in generating and manipulating tunable THz [9–13], infrared [14], and x-rays [15–20]. Among various 2D materials, graphene exhibits a series of unique properties. It features outstanding mechanical strength, remarkable electrical conductivity, high electron mobility, and excellent thermal conductivity [21–24]. Additionally, the energy dispersion of graphene is linear, leading to distinct plasmon dispersion characteristics compared to other 2D electron systems, which exhibit nonlinear energy dispersion [25–29]. Recent experiments have confirmed the nonreciprocal plasmon dispersion in nonequilibrium graphene electron systems, as well as the breaking of Galilean symmetry [30,31]. These findings pave the way for the development of THz light sources based on graphene plasmons.

So far, THz radiation excited by surface plasmons in graphene has been studied both theoretically [32–45] and experimentally [46,47]. Plasmons in graphene, oscillating in the THz range, can be driven by drifting electrons via plasmon instabilities, which are predicted in various structures such as single-layer graphene-based field-effect transistors with a single gate [32–36], grating-gate structures [37,38], double-layer graphene systems [39–42], and graphene nanoribbon arrays [47]. Plasmon instability is a process where a small deviation from the dynamic equilibrium becomes the cause of a further deviation. This process results in the energy transfer from drifting electrons to plasmons, leading to electromagnetic wave radiation [48]. Specifically, in traditional single-gated field-effect transistors based on 2D electron gas (2DEG), there are 2 main types of plasmon instabilities: Dyakonov–Shur [49] and Ryzhii–Satou–Shur instabilities [50]. The former predominantly depends on asymmetric boundary conditions to enhance a plasmon wave at the drain. In contrast, the latter augments a plasmon wave via transit-time effects within the high-electric-field region adjacent to the drain side. Due to the extremely high electron mobility of graphene, these 2 instabilities can be achieved more readily and effectively in graphene-based field-effect transistors [32]. Notably, in dual-grating-gate graphene-based field-effect transistors, both instabilities can be achieved at specific configurations, resulting in marked plasmon amplification [37]. Additionally, researchers have investigated the current-driven plasmon instability in grating-gate graphene-based structures by modulating the carrier density [38]. Such instability has previously been explored in semiconductor 2DEG systems [51,52]. In double-layer graphene systems, 2-stream instability, which is caused by the inconsistent velocities of 2 sets of carriers, has been investigated [39]. Moreover, recent theoretical works have proposed novel mechanisms in graphene [40–45], including non-Cherenkov amplification of THz plasmons in double-layer graphene with direct current [40], as well as the dissipative instability induced by momentum relaxation in single-layer graphene [43]. Experimentally, the researchers have demonstrated current-driven excitation of Dirac plasmons in grating-gate graphene transistor nanostructures, leading to THz radiation amplification up to room temperature [46]. The majority of the aforementioned studies employ the hydrodynamic model to depict the motion of carriers within graphene. However, the hydrodynamic model has limitations in fully describing the carrier distribution in the momentum space, making it impossible to observe certain microscopic plasmon instabilities. A detailed analysis of a representative hydrodynamic model for graphene, as presented in Ref. [32], is introduced in the Supplementary Materials.

Here, we have discovered a novel plasmon instability in single-layer gated graphene by using a method going beyond the hydrodynamic model. The novel plasmon instability has the characteristics of ultra-broadband THz amplification, and its amplification efficiency can be adjusted by the electron concentration and the electron drift velocity. Specifically, we theoretically derive the graphene plasmon dispersion by utilizing both the Boltzmann transport equation and Maxwell’s equations. The former is used to describe the phase-space motion of electrons, and the collision term is treated via the relaxation time approximation. We also investigate the factors influencing plasmon instability and elucidate its underlying origins. It originates from the nonuniformity of the electron–phonon scattering rate across the tilted Fermi surface. Furthermore, we study the amplification process of graphene plasmon using the semi-Lagrangian method [53] and discover the generation of a THz frequency comb. Our results pave the way for developing tunable on-chip THz light sources, amplifiers, and frequency comb generators within integrated THz graphene plasmonic systems.

## Results and Discussion

### Device structure and physical model

**Fig. 1.**
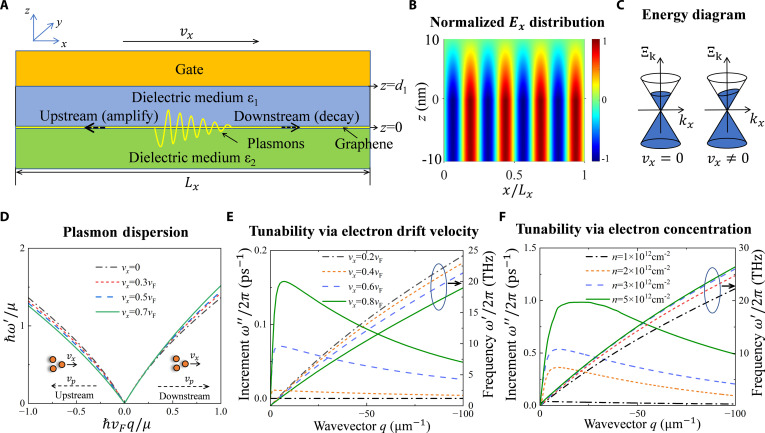
THz amplification induced by graphene plasmon instability in single-layer gated graphene structure. (A) Diagram of our THz amplification system. The graphene is sandwiched by 2 dielectric media, with a metal gate on the upper medium. With drifting electrons, the amplifying plasmons propagate against the electron drift velocity. (B) Normalized distribution of the longitudinal electric field of graphene plasmons. (C) Energy diagrams of graphene without and with drifting electrons. Ξ_k_ is the electron energy. (D) Graphene plasmon dispersions with different drift velocities. The dashed black arrows denote the propagation direction of plasmons, while the straight black arrows signify the drift direction of carriers. (E) Tunability on amplification efficiency and plasmon resonances via electron drift velocity when electron concentration *n* = 1 × 10^12^ cm^−2^. (F) Tunability on amplification efficiency and plasmon resonances via electron concentration when electron drift velocity *v_x_* = 0.5*v*_F_.

The device we used to amplify THz waves is shown in Fig. 1A. Our device is composed of a graphene layer interposed between 2 dielectric media. The top dielectric layer is overlaid by a metal gate. The graphene layer supports surface plasmons. The attractiveness of employing graphene to support surface plasmons lies in the fact that the carrier concentration within graphene can be continuously tuned from nearly zero to as high as 10^13^ cm^−2^ through the application of an external voltage. This important ambipolar doping effect has unveiled the enticing possibility of achieving real-time control of surface plasmons [54].

In the proposed mechanism, the presence of a drift velocity induces a strong asymmetry in the electron–phonon scattering rate across the Fermi surface. This asymmetry leads to electron redistribution in phase space during transport, enhancing local charge concentration perturbations and thereby effectively amplifying the plasmons. To better illustrate our findings, we physically model the system as described below.

Two types of electromagnetic surface waves, namely, the transverse electric (TE) and transverse magnetic (TM) surface modes, are capable of propagating in graphene. Arising from longitudinal charge concentration oscillations, graphene plasmons exhibit a longitudinal electric field component, classifying them as TM modes. In the THz and far-infrared regimes, graphene’s conductivity is dominated by intraband carrier dynamics, which inherently facilitates TM-mode propagation by enabling efficient coupling between electromagnetic fields and charge concentration fluctuations [55]. Here, the Boltzmann transport equation [56–58] is adopted to describe the motion of electrons in phase space:

$$\left(\frac{\partial }{\partial t}+v_{F}\frac{k_{x}}{\left|k\right|}\frac{\partial }{\partial x}-\frac{{\mathrm{eE}}_{x}}{\hbar }\frac{\partial }{\partial k_{x}}\right)f\left(x,\;k,\;t\right)=S_{e-\mathrm{ph}}$$ (1)

Here, *v*_F_ is the Fermi velocity, ***k*** = (*k_x_*, *k_y_*) is the wavevector, *e* is the electron charge, *E_x_* denotes the longitudinal electric field, *ħ* is the reduced Planck’s constant, *f*(*x*,***k***,*t*) is the distribution function of electrons, and *S*_e-ph_ is the collision integral that accounts for electron–phonon scattering. Combined with Maxwell’s equations, the graphene plasmon dispersion is derived:

$$\begin{array}{c} \frac{{\mathrm{ge}}^{2}\mu }{2\pi^{2}\hbar^{2}v_{F}\varepsilon_{0}\left(\varepsilon_{1}\coth\left(\gamma_{1}d_{1}\right)+\varepsilon_{2}\right)}\\ \int_{0}^{\pi }{\left(\omega -{\mathrm{qv}}_{F}\cos\;\theta +\mathrm{i\Gamma}\left(k_{F}\left(\theta \right)\right)\right)}^{-1}\frac{\cos\;\theta -\beta }{{\left(1-\beta \cos\;\theta \right)}^{2}}\mathrm{d\theta}-1=0 \end{array}$$ (2)

Here, *g* = *g*_s_*g*_v_ stands for the degeneracy of electrons, *g*_s_ = 2 is spin degeneracy and *g*_v_ = 2 is the valley degeneracy, *μ* is chemical potential, and *ε*_0_ denotes the permittivity of free space. $\gamma_{1}=\sqrt{\left(q^{2}-\omega^{2}\varepsilon_{1}/c^{2}\right)}\;$is the constant of evanescent decay of the surface wave field in the transverse direction from graphene; *d*_1_ is the height of the top media; *q* and *ω* are the wavevector and angular frequency of surface plasmon, respectively; *k*_F_ is the Fermi wavevector; *Г* is the scattering rate between electron and phonon; and *β* = *v_x_*/*v*_F_ is the relative velocity. The details of theoretical derivation are provided in Methods. By solving the dispersion relation [Eq. (2)] numerically, the complex oscillation frequency of graphene plasmon $\omega =\omega^{\prime }+{\mathrm{i\omega}}^{\prime \prime }$ is obtained. Its real and imaginary parts represent the resonances and increments (amplification efficiency).

### Mechanism of plasmon instability

Figure 1A illustrates the THz amplification due to graphene plasmon instability induced by drift velocity in a single-layer gated graphene system. In the proposed mechanism, plasmons propagating along the drift velocity (downstream plasmons) attenuate gradually, while plasmons propagating against the drift velocity (upstream plasmons) amplify. Figure 1B shows that the normalized electric field of plasmons is sinusoidal along the *x* direction, with its maximum amplitude occurring in the graphene layer (*z* = 0) and decaying in the medium. This reflects the strong confinement of the graphene plasmons. Figure 1C depicts the energy diagrams of graphene without and with electron drift velocity. It can be observed that the Fermi energy is constant without drift velocity. However, the Fermi surface tilts within the Dirac cone when the electrons have a drift velocity, causing the Fermi energy to change with momentum. From Fig. 1D, we observe that the introduction of drift velocity markedly alters the plasmon dispersion, leading to nonreciprocal plasmon propagation that has been experimentally verified [30,31]. Due to the Doppler effect, the downstream plasmon exhibits a blueshift at a fixed wavevector accompanied by an enhanced group velocity. Conversely, the upstream plasmon is redshifted with a reduced group velocity. Furthermore, the results reveal that the frequency shifts become more notable as the drift velocity increases, indicating a direct correlation between the Doppler effect and the frequency of the amplified mode. As shown in Fig. 1E, positive increments occur for upstream plasmon propagation, indicating the amplification in the THz range. The amplification efficiency can be modulated by the electron drift velocity. As the drift velocity gradually increases from 0.2*v*_F_ to 0.8*v*_F_, the amplification efficiency increases from a negative value to 0.16 ps^−1^. Further, the positive increment persists over a broad range of wavevectors, suggesting the characteristic of ultra-broadband amplification. The maximum increment occurs in the long wavelength region, corresponding to several THz. This may imply that the plasmons with several THz will dominate in the amplification process. It is expected to provide a possibility for the scarce THz sources (0.1 to 10 THz). Figure 1F shows that electron concentration is another key factor that can markedly regulate the amplification efficiency. As the electron concentration increases from 1 × 10^12^ cm^−2^ to 5 × 10^12^ cm^−2^, the amplification efficiency increases from about 0.15 ps^−1^ to close to 1 ps^−1^, which means a decrease in propagation distance for THz amplification. We also observe the peak shifts to larger wavevectors. This shift may result in the plasmons with higher frequency becoming dominant.

**Fig. 2.**
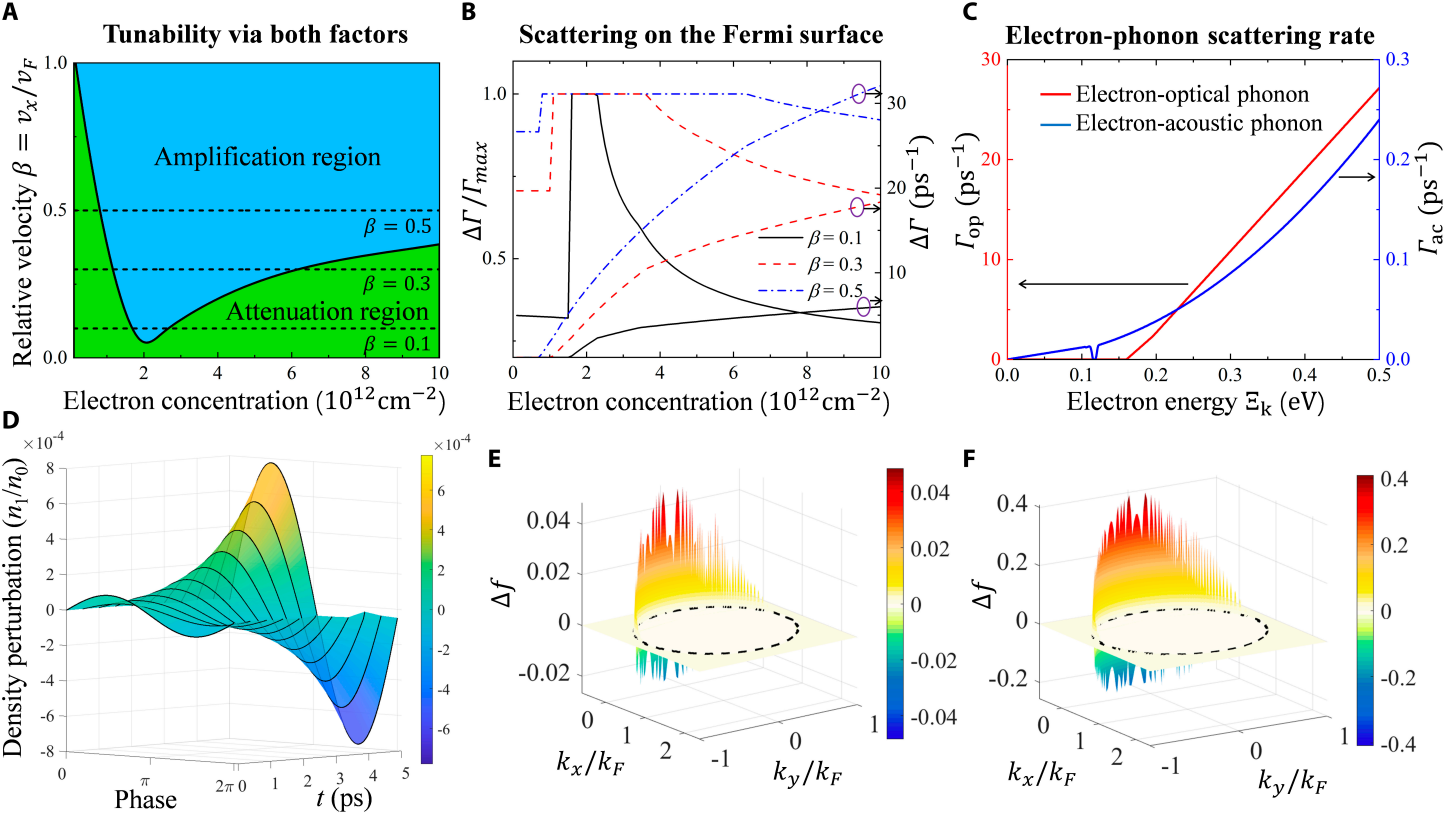
The origin of the graphene plasmon instability. (A) THz amplification region under the combined influence of electron concentration and drift velocity. (B) Difference Δ*Г* between the maximum *Г*_max_ and minimum *Г*_min_ scattering rate on the Fermi surface and the relative difference Δ*Г*/*Г*_max_ in the whole electron concentration region. The electron drift velocities correspond to the 3 dashed lines in (A). (C) Scattering rates of electrons with optical and acoustic phonons when electron concentration *n* = 1 × 10^12^ cm^−2^. (D) Time-dependent evolution of electron concentration perturbation in the wave frame. (E and F) Change in the distribution function at the wave crest relative to its initial state at *t* = 1 and 5 ps, respectively. The black dashed curve indicates the Fermi surface.

To reveal the properties of the instability, we investigate the threshold required for amplification. This analysis explores a broad range of electron concentrations, encompassing typical doping levels for graphene (0.1 to 0.5 eV), and considers drift velocities ranging from zero to the Fermi velocity *v*_F_. Figure 2A shows the region where THz amplification is achieved under the combined influence of electron concentration and electron drift velocity. It indicates that the drift velocity threshold for plasmon instability initially decreases and then increases with increasing electron concentration. The minimum threshold occurs at approximately *n* = 2 × 10^12^ cm^−2^, indicating that only a relatively low drift velocity is needed for amplification at this concentration. This is particularly advantageous for practical applications, as it relaxes the limitation for high electron mobility. To explain this phenomenon, we choose 3 dashed lines in Fig. 2A and plot their maximum scattering rate differences (Δ*Г* = *Г*_max_ − *Г*_min_) and the relative differences Δ*Г*/*Г*_max_ on the Fermi surface. The results are shown in Fig. 2B. In the THz amplification region, the scattering rate differences Δ*Г* are all on the order of 1 and 10 ps^−1^. Meanwhile, the relative differences Δ*Г*/*Г*_max_ are also large, approximately greater than 0.8. This indicates that the key to achieving THz amplification is to create large relative scattering rate differences on the Fermi surface. Further, we investigate the scattering rates of electrons with acoustic and optical phonons. Figure 2C shows that the strength of the electron–optical phonon interactions is much higher than that of the electron–acoustic phonon interactions. Combined with Fig. 2B, we can conclude that the THz amplification is dominated by the electron–optical phonon interactions. We also demonstrate that although the instability is determined jointly by 3 optical phonon modes, the contribution from the phonons near the **K** point dominates due to its higher scattering rate with electrons (see detailed analysis in the Supplementary Materials).

Figure 2D tracks the amplitude evolution of the electron density perturbation over one period, showing an initial decay followed by a sustained growth. To elucidate the amplification mechanism, we examine the evolution of electron momentum-space distribution at the wave crest, as presented in Fig. 2E and F. The results indicate a growing electron clustering in low-scattering-rate regions near the Fermi surface at negative momenta (−*k*_x_), leading to the enhancement of the wave amplitude. Based on these findings, we propose the following explanation. Initially, the drift velocity induces a nonuniform scattering rate distribution across the tilted Fermi surface. Perturbations established in high-scattering-rate regions are rapidly damped, causing the initial wave attenuation. Meanwhile, the electrons are redistributed in the momentum space and accumulate preferentially in low-scattering-rate regions. Although the individual electron velocity is lower than the phase velocity of the wave, the collective motion of the electron cluster synchronizes with the wave crest, thereby continuously delivering energy to the wave. Once the rate of electron cluster formation exceeds the wave-damping rate (i.e., the threshold in Fig. 2A is surpassed), net amplification occurs. The excited plasmon must therefore acquire negative momentum from the clustered electrons through momentum conservation, confirming its upstream nature.

Based on the above physical explanation, we believe that graphene has 2 unique advantages over other plasmonic materials or 2D systems. Specifically, graphene’s linear energy dispersion enables the formation of electron flows moving against the drift, especially under conditions of high drift velocities. This counterflow plays a crucial role in making the amplification of upstream plasmons possible. Additionally, the linear energy dispersion leads to a broad carrier distribution in momentum space, resulting in a large variation in energy at the tilted Fermi surface. This characteristic amplifies the variations in scattering rates across the Fermi surface, thereby enhancing the proposed amplification mechanism.

Compared to the recent dissipative instability in single-layer graphene [43], the instability proposed in this work similarly employs electron drift velocity and momentum relaxation to achieve non-Cherenkov amplification, with similar tunable increments (0.1 to 1 ps^−1^) in the THz range. However, their results differ due to the distinct physical models. The dissipative instability amplifies downstream plasmons with phase velocities near the electron drift velocity, whereas the present mechanism amplifies upstream plasmons and exhibits pronounced sensitivity to momentum-space variations in the relaxation rate. The different theoretical approaches also determine their respective operational conditions. The dissipative instability is valid in the hydrodynamic regime. It requires that the mean free path of electron–electron scattering *l*_ee_ becomes much shorter than that of the electron–phonon scattering and the system dimensions [59]. In contrast, the electron–phonon scattering dominates over the electron–electron scattering in the present mechanism, which means that the mean free path of electron–phonon scattering *l*_e-ph_ is shorter than *l*_ee_. This difference implies that *l*_ee_ markedly affects the applicability of the 2 mechanisms. According to Ref. [60], *l*_ee_ in single-layer graphene varies with carrier density *n* and temperature *T* as *l*_ee_ ∝ *n*^1/2^/*T*^2^. Therefore, in practical high-mobility graphene field-effect transistors, it is possible to switch between the 2 mechanisms by adjusting the gate voltage and temperature. Furthermore, as will be discussed in the subsequent section, the nonlinear dynamics associated with the proposed mechanism offer a promising route toward THz frequency comb generation, potentially extending its utility to high-precision spectroscopy and broadband communications.

### THz amplification and frequency comb

To study the amplification process of graphene plasmon, in particular the influence of nonlinear effects on THz amplification, we take advantage of the semi-Lagrangian method [53] to solve the Boltzmann transport equation [Eq. (1)]. The semi-Lagrangian method solves kinetic equations by backwardly tracing particle trajectories in phase space on a fixed grid. It employs interpolation with time-splitting technique to update the distribution function at each time step, avoiding grid distortion while retaining stability for calculation. The detailed procedures of the semi-Lagrangian method are given in the Supplementary Materials.

Here, we briefly describe the construction of the grids. We set Δ*x* ≈ 5 nm, Δ*k_x_* ≈ 3 μm^−1^, and Δ*k_y_* ≈ 5 μm^−1^, where Δ*x*, Δ*k_x_*, and Δ*k_y_* are the spatial grid steps along *x*, *k_x_*, and *k_y_* directions, respectively. Given that the electron drift velocity is aligned with the *x* direction, the electron distribution function exhibits enhanced broadening in momentum space along *k_x_*. To resolve this anisotropic feature, we employ a smaller grid spacing (Δ*k_x_*) in the *k_x_* direction. A nonuniform temporal step size, Δ*t* ϵ [0.1,5] fs, is implemented, as the nonlinear stage evolution introduces higher harmonics, which require shorter time steps for accurate simulation. Additionally, periodic boundary conditions are imposed on the boundaries along the *x* direction. Multiple simulation results show that the above parameter settings maintain the convergence and stability of the solution. In the amplification process, the longitudinal electric field of graphene plasmons has the following form:$E_{x}=E_{x-\text{initial}}e^{\omega^{\prime \prime }t}e^{-i\omega^{\prime }t}$. Here, *E*_*x*-*initial*_ is the amplitude of the longitudinal electric field at the initial time. It is determined by the applied perturbation and can be calculated by Eq. (7). It is worth mentioning that the saturated electric field strength is independent of the initial perturbation, which is mainly determined by the equilibrium electron concentration and electron drift velocity. (The detailed analyses are provided in Section S4.) So, it is appropriate to introduce a dimensionless parameter *ζ* = ln (*E_x_*/*E_x-initial_*) as the relative amplitude of the electric field to characterize the linear amplification process.

**Fig. 3.**
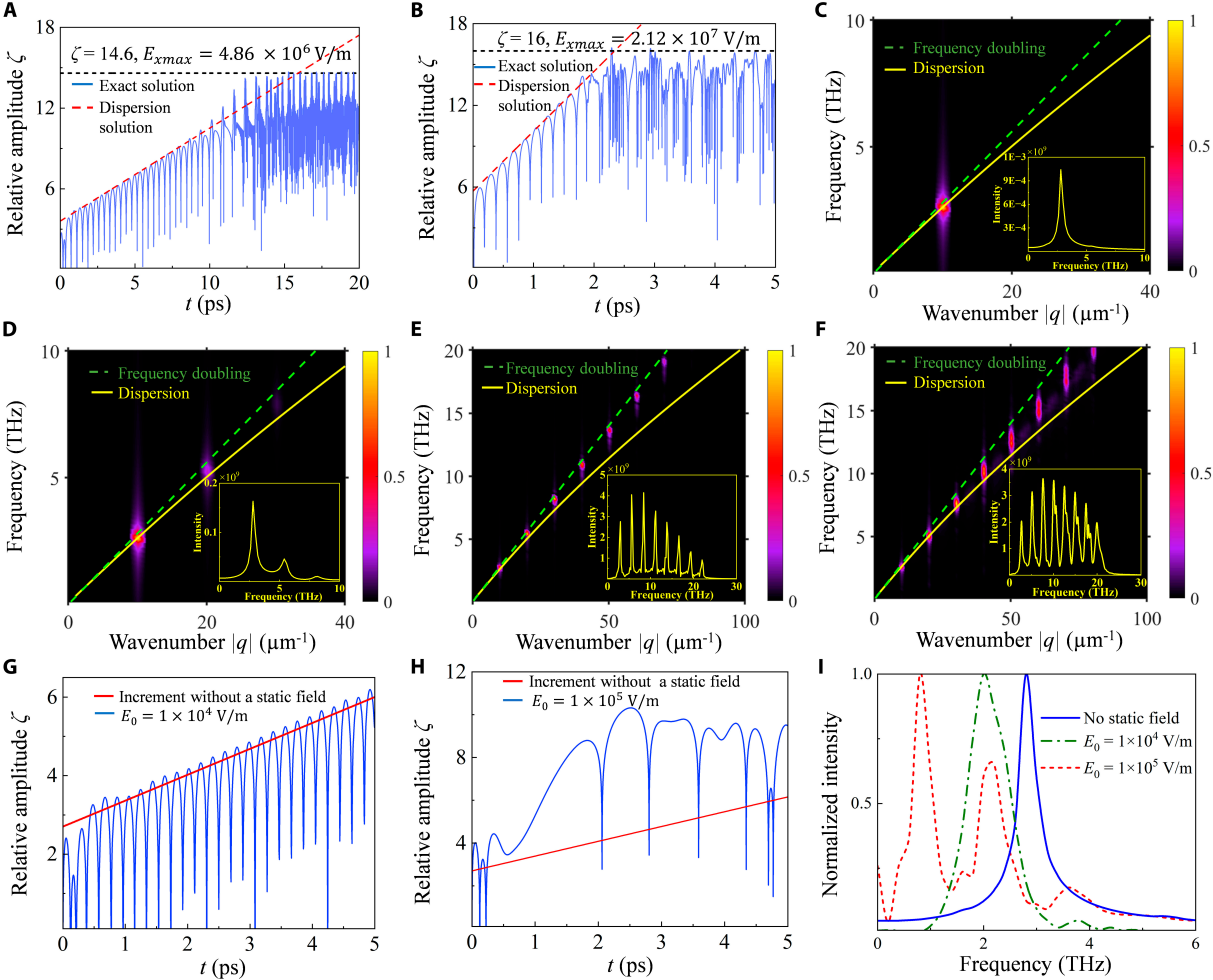
THz amplification under a monochromatic perturbation. (A) The relative amplitude of the longitudinal electric field of the graphene plasmon with electron concentration *n* = 1 × 10^12^ cm^−2^ and drift velocity *v_x_* = 0.7*v*_F_ first amplifies linearly and then gradually becomes saturated. The time required to amplify to saturation is about 12.5 ps. The electric field is amplified by a factor of e^14.6^ (the gain is about 58.28 dB), with the peak up to *E*_*x*max_ = 4.86 × 10^6^ V/m. The red dashed line is the theoretical prediction of dispersion, and the blue curve is the solution of Eq. (1). (B) Relative amplitude of the longitudinal electric field with electron concentration *n* = 5 × 10^12^ cm^−2^ and drift velocity *v_x_* = 0.7*v*_F_. The time required to amplify to saturation is about 2.5 ps. The electric field is amplified by a factor of e^16^ (the gain is about 70.48 dB), with the peak up to *E*_*x*max_ = 2.12 × 10^7^ V/m. (C to F) Fourier transformations of the longitudinal electric field of plasmon in Fig. 2A during time ranges of *t* ϵ [0,5] ps, *t* ϵ [5,10] ps, *t* ϵ [10,15] ps, and *t* ϵ [15,20] ps, respectively. The highlighted points represent plasmon modes, and the mode with the lowest frequency is the fundamental mode. The solid yellow curve is the dispersion solution, and the green dashed line is the frequency-doubling line, whose slope is the ratio of the fundamental mode’s frequency to its wavevector. The inserted figure in each panel is the intensity of the corresponding frequency spectra. (G and H) Relative amplitude of the longitudinal electric field of the plasmon under static electric fields of *E*_0_ = 1 × 10^4^ V/m and *E*_0_ = 1 × 10^5^ V/m, respectively. The slope of the red line represents the increment without a static electric field. The graphene parameters are consistent with those in (A). (I) Spectrum of plasmons during amplification under different static electric field strengths. Each spectrum is obtained by performing a Fourier transform on the plasmon electric field, followed by normalization to its peak value.

In Fig. 3A and B, we introduce a sinusoidal perturbation to the electron concentration whose amplitude is 0.01% of the equilibrium concentration. The results show that the amplification process of graphene plasmons is consistent at different electron concentrations. The relative amplitude of the longitudinal electric field first begins to amplify with a specific increment in accordance with the solution of dispersion and then becomes saturated. The differences brought about by different electron concentrations are mainly reflected in the following 2 aspects. The first is the time required for amplification to saturation, which is about 12.5 and 2.5 ps, respectively. The second is the peak and gain of the electric field of the plasmons. The electric field can peak at 10^6^ and 10^7^ V/m, and the gain can reach about 58 and 70 dB, respectively. Therefore, we believe that high electron concentrations are more favorable for ultrafast and high-gain THz amplification. To capture the changes in the spectrum more clearly, we choose the case where the electron concentration is relatively low, i.e., *n* = 1 × 10^12^ cm^−2^, because in this condition the amplification process is slower and the spectrum does not change too rapidly. Figure 3C shows that in the linear stage, the plasmon frequency is primarily determined by the wavevector of the initial perturbation. During this stage, the plasmon can be approximated as a single-frequency wave, with its amplitude gradually increasing at a constant rate. This mode corresponds to the fundamental mode. When the fundamental mode is amplified adequately, as shown in Fig. 3D and E, higher harmonics are excited, signaling the transition from the linear stage to the nonlinear stage. The nonlinear effect originates from the third term on the left-hand side of Eq. (1), which contains the products of any-order perturbations of the electric field and electron distribution function. These higher harmonics align with the frequency-doubling line, indicating that their frequencies and wavevectors are integer multiples of the fundamental mode. At this time, amplification of the higher harmonics proceeds simultaneously. Notably, these higher-order modes generate a THz frequency comb with a broad spectrum, extending up to 25 THz. Subsequently, these higher harmonics, which initially follow the frequency-doubling line, gradually approach the dispersion, as shown in Fig. 3F. During this process, these frequencies decrease, causing the frequency comb to shift toward the lower frequency band. Meanwhile, these plasmon modes compete with each other and eventually evolve to the dominance of the higher harmonics. In general nonlinearities, the intensity of the higher harmonics should be much less than that of the fundamental mode due to the low conversion rate. However, during the present amplification process, the higher harmonics are gradually enhanced and eventually dominate. In this regard, we infer that this result is due to the property of the ultra-broadband spectrum of the plasmon instability. This means that all the higher harmonics falling within the frequency region of the instability can be amplified.

Additionally, we have investigated the influence of static electric fields *E*_0_ along the −*x* direction on the proposed mechanism. The procedure of applying the static field is detailed in Section S2. Before presenting the results, it is helpful to review the behavior of electron drift velocity in graphene under an external electric field. In field-effect transistors, the drift velocity initially increases approximately linearly with the electric field, with a slope defined by the electron mobility. At higher electric fields, however, the drift velocity saturates, primarily due to the electron–phonon scattering [61]. To simplify our analysis, given this nonlinearity, we fix the drift velocity at *v_x_* = 0.7*v*_F_ over a range of electric field strengths. The simulation results are presented in Fig. 3G to I. Under a relatively weak field *E*_0_ = 1 × 10^4^ V/m, the increment of plasmons shows little deviation from the field-free case (Fig. 3G versus Fig. 3A). However, as the electric field strength increases to *E*_0_ = 1 × 10^5^ V/m (Fig. 3H), a substantial increase in the growth rate is observed, accelerating the transition into the nonlinear stage. This confirms that a stronger static electric field markedly promotes the plasmon amplification. Furthermore, the corresponding plasmon spectra (Fig. 3I) reveal a gradual redshift in the fundamental mode frequency and an obvious enhancement in higher-order harmonics with increasing field strength.

Currently, these results are derived from calculations and analyses performed on ideal monochromatic perturbations. However, it remains to be verified whether the graphene plasmon can guarantee the purity of the spectrum during the THz amplification process in the presence of noise interferences. We define hybridization 1 as consisting of a monochromatic wave at 1.6 THz superimposed on random noise, and hybridization 2 as consisting of 2 monochromatic perturbations whose frequencies are 1.6 and 2.8 THz, respectively. In hybridization 2, the perturbation with 1.6 THz is considered as monochromatic noise. The signal-to-noise ratio (SNR) of hybridizations 1 and 2 are about 5 and 12.5 dB, respectively, according to the formula: SNR = 10lg (*E*_s_/*E*_n_), where *E*_s_ and *E*_n_ are the electric field amplitudes of the monochromatic perturbation and the noise perturbation, respectively.

**Fig. 4.**
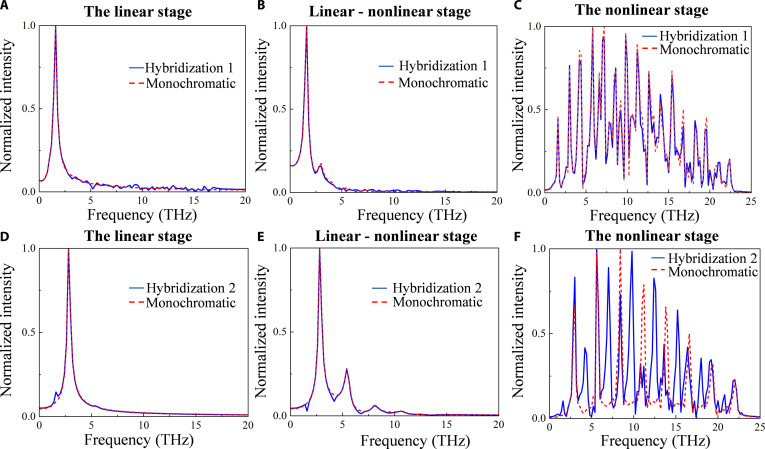
Interference immunity in THz amplification processes. (A to C) Spectra of hybridization 1 and monochromatic perturbations at the linear stage (*t* ϵ [0,5] ps), transition stage from linear to nonlinear stage (*t* ϵ [5,10] ps), and saturation stage (*t* ϵ [10,15] ps), respectively. The SNR of hybridization 1 is about 5 dB. (D to F) Spectra of hybridization 2 and monochromatic perturbations at the linear stage (*t* ϵ [0,5] ps), transition stage from linear to nonlinear stage (*t* ϵ [5,10] ps), and saturation stage (*t* ϵ [10,15] ps), respectively. The SNR of hybridization 2 is about 12.5 dB. The solid blue line represents the hybridization, and the dashed red line represents the monochromatic perturbation. Here, electron concentration *n* = 1 × 10^12^ cm^−2^ and electron drift velocity *v_x_* = 0.7*v*_F_ are adopted. All spectra are obtained through the Fourier transformation of the plasmonic electric field and subsequently normalized to their respective peak values.

From Fig. 4A to C, we can find that in the case of hybridization 1, the influence of noise on the spectrum during amplification is very limited, especially in the linear stage, where the effect due to noise is negligible. Figure 4D to F shows that the monochromatic noise in hybridization 2 can alter the spectrum markedly as higher harmonics are generated and amplified in the nonlinear stage. Especially when the electric field is saturated, the effect is remarkable, producing a superposition of 2 sets of THz frequency combs. Although monochromatic noise changes the results of the frequency comb in the nonlinear stage, it has little effect on the spectrum of the linear stage. From the results of the 2 cases, it is clear that the monochromatic noise with a higher SNR instead has a larger effect on the spectrum of the monochromatic perturbation than the random noise with a lower SNR. We argue that the random noise contains many frequency components. The components falling within the amplification region are limited and their amplitudes are not enough, so the effect on the spectrum of monochromatic perturbation is negligible. Compared to random noise, monochromatic noise contains only a single frequency component that falls within the amplification region, which makes it possible to alter the spectrum markedly. To summarize, we believe that the plasmon instability is highly resistant to interference and maintains the purity of the spectrum well before the electric field is amplified to saturation during THz amplification. This feature is advantageous for THz amplifiers.

We believe that the novel graphene plasmon instability has 2 potential applications. The first application is an ultra-broadband THz amplifier. By controlling the electron transit time to maintain operation in the linear stage, we can suppress the generation of higher harmonics, ensuring the amplification of the THz wave and the purity of its spectrum. For example, when we set the electron drift velocity *v_x_* = 0.7*v*_F_ and the electron concentration *n* = 1 × 10^12^ cm^−2^ or *n* = 5 × 10^12^ cm^−2^, the linear stage time of graphene plasmon amplification is within 10 and 2 ps, respectively. Thus, the device’s operational length would be on the order of micrometers and submicrometers. The second application is the THz frequency comb generator, which requires a longer electron transit time to facilitate frequency multiplication and amplification in the nonlinear stage.

### Experimental perspectives

To experimentally verify the THz amplification mechanism investigated in this paper, here we analyze the challenges and the corresponding possible solutions in the following aspects.

Firstly, to achieve precise control over carrier concentration and drift velocity in graphene, the fabrication of graphene-based field-effect transistor structures with grating gates is a feasible option [37,46]. In these structures, the carrier concentration can be modulated by the gate voltage, and the source–drain voltage drives the electrons to accelerate until saturation. The output characteristic curve of the device characterizes carrier mobility and drift velocity. Secondly, realizing the proposed mechanism hinges on large scattering rate differences across the Fermi surface, which is easier to achieve at high drift velocities. Thus, fabricating graphene-based devices with ultrahigh carrier mobility is experimentally critical yet challenging. To achieve this objective, optimization can focus on 3 key aspects: monolayer graphene preparation, encapsulating dielectric selection, and electrode contact engineering. Among fabrication techniques for monolayer graphene, mechanical exfoliation and chemical vapor deposition (CVD) are dominant. The former offers high-quality samples at low cost, while the latter enables large-area synthesis [62]. Given the required micrometer-scale device dimensions, mechanical exfoliation is preferable for graphene preparation. For dielectric encapsulation, hexagonal boron nitride (hBN)/graphene/hBN heterostructures are ideal due to graphene’s ultrahigh mobility in hBN. This heterostructure exhibits low charge carrier inhomogeneity and ballistic transport over micrometer lengths at low temperatures [63]. Most studies fabricate such heterostructures via mechanical exfoliation and layer-by-layer transfer [64]. Critically, optimizing fabrication methods and electrical contacts has yielded devices with room-temperature mobility as high as 140,000 cm^2^/Vs [65]. This is achieved through van der Waals assembly of heterostructures, where 1D electrode contacts are formed by etching the top-layer hBN to expose graphene edges.

After fabricating the devices, exciting the graphene plasmon successfully is another challenge. In general, there are 2 approaches. The first is self-excited oscillation. Thermal noise distributed in the environment causes carriers in graphene to generate density perturbations, thus exciting the graphene plasmon [66]. In this way, 2 modes can be excited at the same time, i.e., downstream and upstream plasmons. The upstream plasmon can be amplified, while the downstream plasmon is attenuated when the plasmon instability studied in the manuscript is excited. The nature of this method lies in the fact that the thermal noise contains a large number of quasiparticles, which are plasmons with rich frequency information. Secondly, the graphene plasmon can be excited by external electromagnetic waves through momentum matching, which usually can be realized by gratings, nano-antennas, and near-field couplers [67]. This approach can be used to excite upstream plasmons by adjusting the coupling structure and the angle of incidence. It is also expected to produce coherent THz radiation.

To summarize, although it will be challenging to experimentally validate our results, some outstanding works prove the feasibility of carrying out the experiments to the best of our knowledge. This gives us strong expectations for future experimental observations of the mechanism by researchers.

## Conclusion

We have demonstrated a novel plasmon instability and achieved ultra-fast, high-gain THz amplification in a single-layer gated graphene structure. The instability originates from the nonuniformity of the electron–phonon scattering rate across the tilted Fermi surface, which leads to electron clustering in phase space and upstream plasmon amplification. Specifically, the longitudinal electric field of graphene plasmons can be amplified to 10^7^ V/m within 2.5 ps. The plasmon instability has an ultra-broadband amplification spectrum of up to 25 THz and an extremely high increment on the order of 1 ps^−1^ at high electron concentrations (*n* ~ 10^13^ cm^−2^). Especially, the peak of increment appears in the long wavelength region where the wavevectors correspond to resonances exactly below 10 THz. It provides a promising way for preparing the crucial and scarce band of THz sources. Additionally, although the tunability on plasmon instability implies that high drift velocities are more conducive to achieving THz amplification, by adjusting the carrier density, relatively low drift velocities also support the realization of this mechanism. It reduces the requirements for high electron mobility. We also found the generation of a THz frequency comb whose harmonic intensities are comparable to that of the fundamental mode in the nonlinear stage of the plasmon amplification process. This frequency comb will gradually move to a lower THz band eventually below 20 THz as the process evolves. The hybridization results elucidate the excellent immunity to interference of the instability. In summary, our findings suggest potential applications for highly integrated, on-chip, tunable THz sources, amplifiers, and frequency comb generators based on graphene plasmons.

## Methods

The magnetic fields of the surface plasmon have the following form [68]:

$$H_{y}=\left\{\begin{array}{c} \left(A_{1}e^{-\gamma_{1}z}+A_{2}e^{\gamma_{1}z}\right)e^{\mathrm{iqx}-\mathrm{i\omega t}},0<z<d_{1}\\ \left(A_{3}e^{-\gamma_{2}z}+A_{4}e^{\gamma_{2}z}\right)e^{\mathrm{iqx}-\mathrm{i\omega t}},z<0 \end{array}\right.$$ (3)

where *A*_1_…*A*_4_ are coefficients; *q* and *ω* are the wavevectors and angular frequency of surface plasmon, respectively; and *c* is the speed of light in vacuum. Here, $\gamma_{j}=\sqrt{\left(q^{2}-\omega^{2}\varepsilon_{j}/c^{2}\right)}$ is the constant of evanescent decay of the surface wave field in the transverse direction from graphene, where *j* = 1, 2 refers to layers 1 and 2. *d*_1_ is the height of the top media, and the bottom media can be considered to be semi-infinite. The plasmon electric field components of the TM modes can be derived by introducing Eq. (3) into Maxwell’s equation for dielectric media

$$\nabla \times H=J+\frac{\partial }{\partial t}D,$$ (4)

where ***D*** = *ε*_0_*ε_j_****E*** is the electric displacement vector in isotropic media and *ε*_0_ denotes the permittivity of free space. In the dielectric media, the current density ***J*** = 0. The derivative of time can be expressed in the form of harmonics, i.e., ∂/∂t=−iω. Therefore, the plasmon electric field of the TM modes ***E*** *=* (*E_x_*, *E_z_*) can be expressed as:

$$E=\frac{i}{{\omega \varepsilon }_{0}\varepsilon_{j}}\nabla \times H.$$ (5)

To eliminate the coefficients *A*_1_...*A*_4_, we introduce the boundary conditions into Eq. (5),

$$\begin{array}{c} \begin{array}{l} z=d_{1}\to E_{x1}=0\\ z=0\to \left\{\begin{array}{c} E_{x1}=E_{x2}\\ D_{z1}-D_{z2}=-e\left(n\left(x,\;t\right)-n_{0}\right), \end{array}\right.\\ z=-\infty \to E_{x2}=0 \end{array} \end{array}$$ (6)

where *e* is the electron charge, *n*(*x*,*t*) is the electron concentration, and *n*_0_ is the equilibrium concentration. Then, we get the plasmon electric field at the graphene–dielectric interface (*z* = 0):

$$E_{x}=\frac{\mathrm{ie}\left(n\left(x,\;t\right)-n_{0}\right)}{\varepsilon_{0}\left(\varepsilon_{1}\coth\left(\gamma_{1}d_{1}\right)+\varepsilon_{2}\right)}.$$ (7)

Equation (7) indicates that the longitudinal electric field of surface plasmons is directly related to the electron concentration perturbation on the graphene surface. Here, the Boltzmann transport equation, which describes the motion of electrons, is adopted to derive the electron concentration on the graphene surface:

$$\left(\frac{\partial }{\partial t}+v_{F}\frac{k_{x}}{\left|k\right|}\frac{\partial }{\partial x}-\frac{{\mathrm{eE}}_{x}}{\hbar }\frac{\partial }{\partial k_{x}}\right)f\left(x,\;k,\;t\right)=S_{e-\mathrm{ph}},$$ (8)

where *v*_F_ is the Fermi velocity, *ħ* is the reduced Planck’s constant, *S*_e-ph_ is the collision integral that accounts for electron–phonon scattering, and *f*(*x,**k**,t*) is the distribution function of electrons. Considering the collision term *S*_e-ph_, the most straightforward approach is to incorporate the relaxation time approximation:

$$S_{e-\mathrm{ph}}=\Gamma \left(f\left(x,\;k,\;t\right)-f_{0}\left(k\right)\right),$$ (9)

where *f*_0_(***k***) is the equilibrium distribution and *Γ* is the electron–phonon scattering rate. In this description, the electron concentration can be obtained by integrating the distribution function over the momentum space:

$$n\left(x,\;t\right)=\frac{g}{{\left(2\pi \right)}^{2}}\iint f\left(x,\;k,\;t\right)dk_{x}dk_{y},$$ (10)

where *g* stands for the degeneracy of electrons. In graphene, *g* = *g*_s_*g*_v_, where *g*_s_ = 2 represents spin degeneracy and *g*_v_ = 2 indicates the valley degeneracy. By combining Eqs. (7) and (10), the plasmon electric field of graphene is obtained:

$$E_{x}=\frac{\mathrm{ie}}{\varepsilon_{0}\left(\varepsilon_{1}\coth\left(\gamma_{1}d_{1}\right)+\varepsilon_{2}\right)}\frac{g}{{\left(2\pi \right)}^{2}}\iint \left(f\left(x,\;k,\;t\right)-f_{0}\left(k\right)\right)dk_{x}dk_{y}$$ (11)

So far, we have obtained 2 basic equations for deriving the dispersion, which are Eqs. (8) and (11). We will perform a small perturbation analysis on the distribution function *f*(*x*,***k***,*t*) and the longitudinal electric field *E_x_* to obtain the dispersion. We believe that the small perturbation is a plane-like wave.

$$\begin{array}{c} f\left(x,\;k,\;t\right)=f_{0}\left(k\right)+f_{1}e^{i\left(\mathrm{qx}-\omega t\right)}\\ E_{x}=E_{1x}e^{i\left(\mathrm{qx}-\omega t\right)} \end{array}$$ (12)

Here, *f*_0_(***k***) is the drifting Fermi distribution function and it can be expressed as:

$$f_{0}\left(k\right)={\left(\exp\left(\frac{\Xi_{k}-p_{x}v_{x}-\mu }{k_{B}T}\right)+1\right)}^{-1},$$ (13)

where Ξ_k_ = *ħv*_F_|***k***| is the energy of electrons in graphene, *p_x_* = *ħk_x_* denotes the momentum of the electron along the *x* direction, *v_x_* is the electron drift velocity, *μ* is the chemical potential, and *k*_B_ is the Boltzmann constant. *f*_1_ and *E*_1*x*_ are the amplitude of the perturbations of electron distribution and the longitudinal electric field, respectively. In the graphene system with drifting electrons, the chemical potential *μ* is related to both temperature *T* and velocity *v_x_*.

$$\mu =E_{F}\left(1-\frac{\pi^{2}}{6}{\left(\frac{T}{T_{F}}\right)}^{2}\right){\left(1-\beta^{2}\right)}^{\frac{3}{4}},$$ (14)

where *E*_F_ = *ħv*_F_*k*_F_ is the Fermi energy, *T*_F_ = *E*_F_/*k*_B_ is the Fermi temperature, and *β* = *v_x_*/*v*_F_ is the relative velocity. To obtain the perturbation of electron distribution function *f*_1_, electron concentration *n*_1_, and longitudinal electric field *E*_1*x*_, we introduce Eq. (12) into Eqs. (8), (10), and (11), and after linearization, we can get

$$f_{1}=\frac{i}{\omega -{\mathrm{qv}}_{F}k_{x}/\left|k\right|+\mathrm{i\Gamma}}\frac{{\mathrm{eE}}_{1x}}{\hbar }\frac{\partial f_{0}}{\partial k_{x}}.$$ (15)

$$n_{1}=\frac{g}{{\left(2\pi \right)}^{2}}\iint f_{1}dk_{x}dk_{y}$$ (16)

$$E_{1x}=\frac{\mathrm{ige}\iint f_{1}dk_{x}dk_{y}}{{\left(2\pi \right)}^{2}\varepsilon_{0}\left(\varepsilon_{1}\coth\left(\gamma_{1}d_{1}\right)+\varepsilon_{2}\right)}=\frac{{\mathrm{ien}}_{1}}{\varepsilon_{0}\left(\varepsilon_{1}\coth\left(\gamma_{1}d_{1}\right)+\varepsilon_{2}\right)}$$ (17)

From Eqs. (15) to (17), we find that these equations form a closed set. Therefore, the dispersion can be derived by combining them and eliminating the perturbation term. First, we substitute Eq. (15) into Eq. (16) to eliminate the perturbation term of the distribution function *f*_1_:

$$n_{1}=\frac{{\text{igeE}}_{1x}}{{\left(2\pi \right)}^{2}\hbar }\iint \frac{1}{\omega -{\mathrm{qv}}_{F}k_{x}/\left|k\right|+\mathrm{i\Gamma}}\frac{\partial f_{0}}{\partial k_{x}}dk_{x}dk_{y}.$$ (18)

After that, we derive the dispersion by combining Eqs. (17) and (18):

$$\begin{array}{c} \frac{{\mathrm{ge}}^{2}}{{\left(2\pi \right)}^{2}\hbar \varepsilon_{0}\left(\varepsilon_{1}\coth\left(\gamma_{1}d_{1}\right)+\varepsilon_{2}\right)}\\ \iint \frac{1}{\omega -{\mathrm{qv}}_{F}k_{x}/\left|k\right|+\mathrm{i\Gamma}}\frac{\partial f_{0}}{\partial k_{x}}dk_{x}dk_{y}+1=0. \end{array}$$ (19)

Equation (19) is an important result of this paper. However, the complicated integral term prevents us from further calculation and analysis. So, we propose some approximate methods to make it simple and computable. First, to deal with the derivative term ∂*f*_0_/∂*k_x_*, it is convenient to employ a reasonable approximation for the distribution function. In this study, the Fermi energy of graphene is controlled by the gate on the order of 0.1 eV. It will result in graphene having a Fermi temperature much higher than room temperature. Therefore, at low temperatures or even room temperature, electrons occupy almost all states with energies less than or equal to the Fermi energy, which is represented mathematically as:

$$f_{0}=1-\Theta \left(\hbar v_{F}\left|k\right|-\hbar k_{x}v_{x}-\mu \right),$$ (20)

where Θ(*x*) stands for Heaviside step function. So, the derivative term can be approximated as:

$$\begin{array}{c} \frac{\partial f_{0}}{\partial k_{x}}=-\delta \left(\hbar v_{F}\left|k\right|-\hbar k_{x}v_{x}-\mu \right)\left(\hbar v_{F}\frac{k_{x}}{\left|k\right|}-\hbar v_{x}\right)=\\ -\delta \left(\left|k\right|-k_{x}\beta -\frac{\mu }{\hbar v_{F}}\right)\left(\frac{k_{x}}{\left|k\right|}-\beta \right), \end{array}$$ (21)

where δ(*x*) represents the Dirac delta function. Then, introducing Eq. (21) into Eq. (19), and transforming the rectangular coordinate system into a polar coordinate system, for simplicity, we define *k* = |***k***|, i.e., *k_x_* = *k*cos*θ*, *k_y_* = *k*sin*θ*, and d*k_x_*d*k_y_* = *k*d*k*d*θ*. Equation (19) becomes

$$\begin{array}{c} \frac{{\mathrm{ge}}^{2}}{{\left(2\pi \right)}^{2}\hbar \varepsilon_{0}\left(\varepsilon_{1}\coth\left(\gamma_{1}d_{1}\right)+\varepsilon_{2}\right)}\\ \iint \frac{\delta \left(k-k_{F}\left(\theta \right)\right)}{\omega -{\mathrm{qv}}_{F}\cos\;\theta +\mathrm{i\Gamma}\left(k\right)}\frac{\cos\;\theta -\beta }{1-\beta \cos\;\theta }kdkd\;\theta -1=0, \end{array}$$ (22)

where *k*_F_(*θ*) = *μ*/*ħv*_F_(1 − *β*cos*θ*) is the Fermi wavevector in the presence of drift velocity. By continuing to integrate the variable *k*, we can easily obtain:

$$\begin{array}{c} \frac{{\mathrm{ge}}^{2}\mu }{2\pi^{2}\hbar^{2}v_{F}\varepsilon_{0}\left(\varepsilon_{1}\coth\left(\gamma_{1}d_{1}\right)+\varepsilon_{2}\right)}\\ \int_{0}^{\pi }{\left(\omega -{\mathrm{qv}}_{F}\cos\;\theta +\mathrm{i\Gamma}\left(k_{F}\left(\theta \right)\right)\right)}^{-1}\frac{\cos\;\theta -\beta }{{\left(1-\beta \cos\;\theta \right)}^{2}}d\;\theta -1=0. \end{array}$$ (23)

Equation (23) is the simplified dispersion of the gated graphene system. From it, we observe that the scattering between electrons and other particles such as phonons occurring on the Fermi surface is the major contribution. In this study, the scattering between electrons and phonons is considered. In detail, we focus on intrinsic electron–phonon interactions in graphene, including the contributions of acoustic phonons and optical phonons.

According to the previous works [69–71], the scattering rate between electrons and acoustic phonons can be expressed as

$$\begin{array}{c} \Gamma_{\mathrm{ac}}\left(k_{x},\;k_{y}\right)=\frac{\Omega \left(k_{x},\;k_{y}\right)}{1-f\left(k_{x},\;k_{y}\right)}\int d\theta \left(1-\cos\;\theta \right)\times \\ \sum_{a,p}D_{a}^{p}\left(\theta \right)\left(N_{q}^{a}+\frac{1}{2}-\frac{p}{2}\right)\left[1-f\left(k_{x},k_{y}\right)\right]. \end{array}$$ (24)

Here, Ω = Ξ_k_^2^/4*πħ*^3^*v*_F_^3^*ρ*_m_, *ρ*_m_ denotes mass density, *a* = LA(TA) indicates the longitudinal (transverse) acoustic phonon, and *p* = ±1, with *p* = +1 indicating the absorption process and *p* = −1 indicating the emission process. $D_{a}^{p}$ = *F_a_*$T_{a}^{p}$, *F*_TA_ = 1/2*v*_TA_*B*^2^, and *F*_LA_ = 1/*v*_LA_(*E*_1_^2^cos^2^(*θ*/2)+*B*^2^/2). *E*_1_ is the screened deformation potential for LA phonons, and *B* is related to gauge field. $T_{a}^{p}=K_{a}^{p}Q_{a}^{p}/d_{a}^{p}$, where $K_{a}^{p}$ = 1 + 2*p_y_v*_a_/*v*_F_, $Q_{a}^{p}$ = 2*y,* $d_{a}^{p}$ = 1 − 2*p_y_v*_a_/*v*_F_, and *y* = sin(*θ*/2). *N*_q_^a^ is the Bose–Einstein distribution function of acoustic phonons.

For optical phonons [72,73], 3 distinct optical phonon modes play a crucial and substantial role in the inelastic scattering of electrons within graphene. The initial 2 pertinent modes are the longitudinal optical (LO) and transverse optical (TO) phonons, which possess wave vectors that are in close proximity to the **Γ** point. These phonons have energies of *ħω*_1_ = *ħω*_2_ = 196 meV. Moreover, it is of great significance to take into account the zone boundary phonons with an energy of *ħω*_3_ = 160 meV, which are located near the **K** point and are accountable for the intervalley processes. For the sake of conciseness, we designate these modes as *η* = 1(**Γ**-LO), 2(**Γ**-TO), and 3(**K**). The scattering rate of optical phonons can be written as:

$$\begin{array}{c} \Gamma_{\mathrm{op}}\left(k_{x},\;k_{y}\right)=\sum_{\eta }\frac{D_{\eta }^{2}\left(N_{\eta }+1/2\mp 1/2\right)}{2\hbar^{2}v_{F}^{2}\rho_{m}\omega_{\eta }}\left(\Xi_{k}\pm \hbar \omega_{\eta }\right) \end{array}$$ (25)

Here, *D*_1_ = *D*_2_ = 14.1 eV/Å, *D*_3_ = 25.6 eV/Å are the optical deformation potentials of graphene and *N*_η_ is the Bose–Einstein distribution function of optical phonon. Thus, we obtain the total scattering rate between electrons and phonons

$$\Gamma \left(k_{x},\;k_{y}\right)=\Gamma_{\mathrm{ac}}\left(k_{x},\;k_{y}\right)+\Gamma_{\mathrm{op}}\left(k_{x},\;k_{y}\right).$$ (26)

Combining Eqs. (26) and (23), the dispersion can be numerically solved.

## Data Availability

All the data are included within the article and its Supplementary Materials.
