# Supplementary material for: Terahertz Amplification Induced by Electron–Phonon Interactions in Gated Graphene Plasmonic System
Source: Research (Wash D C). 2025 Dec 3;8:1023. doi: 10.34133/research.1023 (PMC12673019; doi:10.34133/research.1023)
Supplement: Supplementary 1 — Supplementary Text Figs. S1 to S3 [file research.1023.f1.docx]

**Supplementary Materials for**

**Terahertz amplification induced by electron-phonon interactions in gated graphene plasmonic system**

Zijian Qiu,^1,2^ Shengpeng Yang,^1,2^* Sunchao Huang,^1,2^ Shaomeng Wang,^1,2†^

Ping Zhang,^1,2^ and Yubin Gong^3,1,2§^

^1^National Key Laboratory of Science and Technology on Vacuum Electronics, School of Electronic Science and Engineering, University of Electronic Science and Technology of China

^2^Terahertz Radiation and Application Key Laboratory of Sichuan Province, Chengdu, 611731, China.

^3^Yangtze Delta Region Institute (Quzhou), University of Electronic Science and Technology of China.

*Contact author: [syang@uestc.edu.cn](mailto:syang@uestc.edu.cn)

^†^Contact author: [wangsm@uestc.edu.cn](mailto:wangsm@uestc.edu.cn)

^§^Contact author: [ybgong@uestc.edu.cn](mailto:ybgong@uestc.edu.cn)

This supplementary document consists of three sections. The first section provides the theoretical framework of the hydrodynamic model and presents its derived dispersion relation. Through comparison with the kinetic theory developed in the paper, we conclude that the hydrodynamic model fails to obtain the plasmon instability due to the omission of the momentum-space distribution. The second section elaborates on the semi-Lagrangian method for solving the Boltzmann equation in our simulations. In this part, we summarized the method’s fundamental principle, specific operation procedures, and main characteristics. The third section elaborates on the influence of the three most significant optical phonon modes in graphene on plasmon instability. By individually analyzing the contribution of each phonon mode to the plasmon growth rate, we characterize their respective impacts. Among them, the optical phonon mode near the **K** point plays a dominant role in the amplification process. The last section mainly analyzes the parameters that affect the saturated plasmon electric field. It’s shown that the saturation of oscillating electron concentration is the main factor, which can be adjusted by the equilibrium electron concentration and electron drift velocity.

**1 Hydrodynamic Model**

**1.1 Theory Derivation**

In the hydrodynamic model, the motion of free electrons in graphene is described by the Euler equation [S1]:

 (1)

Here *v_x_* is the electron drift velocity, *v*_F_ is the Fermi velocity, *β* is the relative velocity, *n* represents the electron concentration, *e* is the elementary charge, *E*_1_ denotes the electric field induced by the fluctuations of the electron concentration, *ħ* is the reduced Planck's constant, *n*_0_ and *v*_0_ are respectively the electron concentration and drift velocity in the equilibrium state, and *τ* is the electron momentum relaxation time. Meanwhile, the system also satisfies the continuity equation:

$-e\frac{\partial n}{\partial t}+\frac{\partial J_{x}}{\partial x}=0$, (2)

where *J_x_ = -env_x_* is the current density.

To obtain the dispersion, we will perform a small perturbation analysis on the electron concentration *n*(*x*,*t*), the drift velocity *v_x_*(*x*,*t*) and the current density *J_x_*(*x*,*t*):

$\begin{matrix} n(x,t)=n_{0}+n_{1}\exp(i(qx-\omega t)) \\ v_{x}(x,t)=v_{0}+v_{1}\exp(i(qx-\omega t)) \\ J_{x}(x,t)=-en_{0}v_{0}-e(n_{1}v_{0}+n_{0}v_{1})\exp(i(qx-\omega t)) \end{matrix}$ (3)

After linearizing Eqs. (1) and (2) by Eq. (3), we obtain:

$-i\omega n_{1}+iqv_{0}n_{1}+iqn_{0}v_{1}=0$. (4)

Eliminating the equilibrium terms of electron concentration *n*_0_ and velocity *v*_0_ in Eq. (4) and using the linearization for the current density *J_x_*= *J*_0_+ *J*_1_, we will obtain an equation only containing the perturbation terms of the current density and electric field. According to Ohm's Law, *J*_1_ = *σ_g_E*_1_, where *σ_g_* is the graphene’s conductivity, we get:

. (5)

For the structure depicted in Figure 1a, the magnetic fields of the surface plasmon have the following form [S2]:

$H_{y}=\left\{\begin{matrix} (A_{1}e^{-\gamma_{1}z}+A_{2}e^{\gamma_{1}z})e^{iqx-i\omega t},0<z<d_{1} \\ (A_{3}e^{-\gamma_{2}z}+A_{4}e^{\gamma_{2}z})e^{iqx-i\omega t},z<0 \end{matrix}\right.$, (6)

where *A*_1_…*A*_4_ are coefficients, *q* and *ω* are the wavevectors and angular frequency of surface plasmon, respectively, *c* is the speed of light in vacuum. Here$\gamma_{j}=\sqrt{(q^{2}-\omega^{2}\varepsilon_{j}/c^{2})}$is the attenuation constant of the surface wave, where *j* = 1, 2 refers to layers 1 and 2. *d*_1_ is the height of the top media, and the bottom media can be considered to be semi-infinite. The electric field components of the TM modes can be derived by introducing Eq. (6) into Maxwell’s equation for dielectric media

$\nabla\times H=J+\frac{\partial}{\partial t}D$, (7)

where ***D*** = *ε*_0_*ε_j_****E*** is the electric displacement vector in isotropic media and *ε*_0_ denotes the permittivity of free space. In the dielectric media, the current density ***J*** = 0. The derivative of time can be expressed in the form of harmonics, i.e.,$\partial/\partial t=-i\omega$. Therefore, we can get expressions for the longitudinal electric field components:

$E_{x}=-\frac{i}{\omega\varepsilon_{0}\varepsilon_{a}}\frac{\partial}{\partial z}H_{y}$, (8)

where *E_x_* is the *x* component of the electric field. To eliminate the coefficients *A*_1_...*A*_4_, we introduce the boundary conditions into Eq. (8),

$\begin{matrix} z=d_{1}\to E_{x1}=0 \\ z=0\to\left\{\begin{matrix} E_{x1}=E_{x2} \\ H_{y1}-H_{y2}=-\sigma_{g}E_{x1} \end{matrix}\right. \\ z=-\infty\to E_{x2}=0 \end{matrix}$, (9)

Then, we get the dispersion of the gated graphene system:

$\frac{\varepsilon_{1}}{\gamma_{1}}\coth(\gamma_{1}d_{1})+\frac{\varepsilon_{2}}{\gamma_{2}}+i\frac{\sigma_{g}}{\omega\varepsilon_{0}}=0$. (10)

After combining Eqs. (5) and (10), the dispersion of the system under the hydrodynamic description can be obtained.

**1.2 Results**

In the analysis of the hydrodynamic plasmon dispersion, we set the electron concentration as *n* = 1×10^12^ cm^-2^, and set *τ* to be the mean of the electron momentum relaxation time on the Fermi surface.


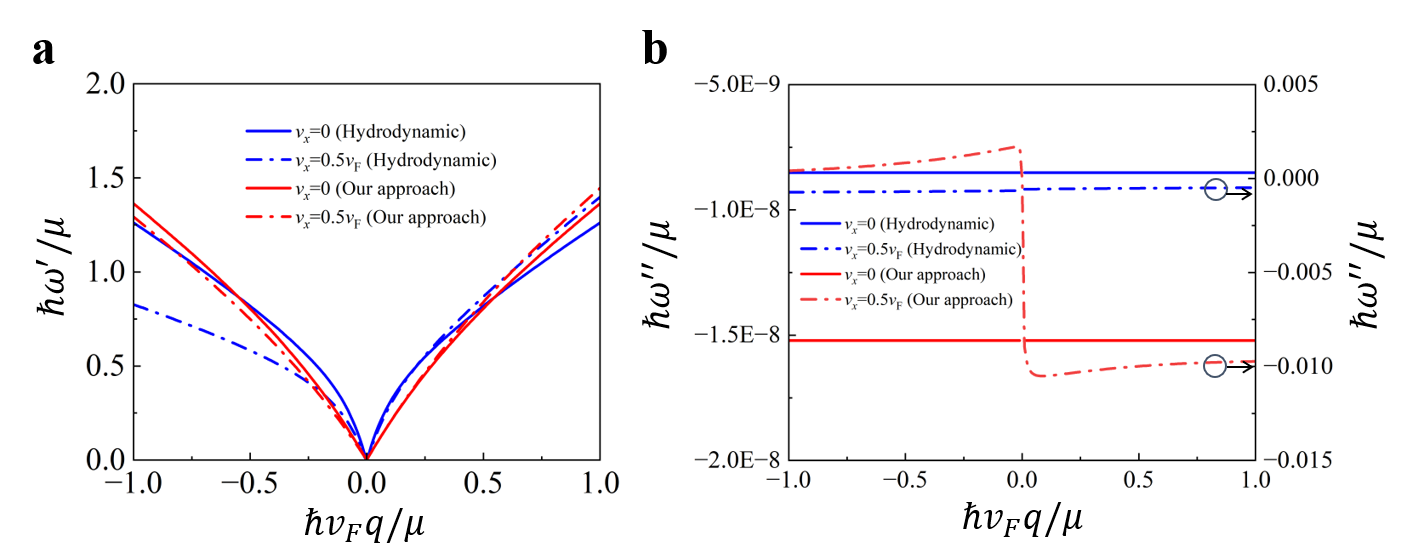


**Figure S1. Dispersions of the gated single-layer graphene system under the previous hydrodynamic model and the approach of this work (Eq. (2) in the paper).** **(a)** Effect of drift velocity on dispersions. The blue curves are the results of the hydrodynamic model and the red curves are the results of our approach. The solid lines represent static systems (*v_x_*=0), and the dotted lines denote the drifting systems (*v_x_*=0.5*v*_F_). **(b)** Imaginary part of the frequency of graphene plasmon, i.e., increment.

Figure S1 shows the comparison of the hydrodynamic model and our approach. From Figure S1a, we can see that the dispersions obtained by both theories are symmetrical without drift velocity, and the differences between them are not significant. However, when the drift velocity is introduced into the system, although the results of the two theories both maintain the characteristic of non-reciprocity, the dispersions are different. Under the hydrodynamic model, the electron drift velocity will more significantly change the dispersion, especially for the upstream plasmon, whose phase velocity is significantly reduced. Furthermore, Figure S1b indicates that under the hydrodynamic model, the imaginary part of the plasmon oscillation is always negative, which implies the disappearance of instability. However, in our approach, the imaginary part of the upstream plasmon changes from negative to positive under a certain drift velocity bias, suggesting the occurrence of instability. This implies that the hydrodynamic model is inadequate in describing microscopic plasmon instabilities. We believe that this result is inextricably linked to the fact that the hydrodynamic model ignores the distribution and transportation of electrons in momentum space.

**2 Semi-Lagrangian Method**

Here, we introduce the technical implementation of the semi-Lagrange method, which is the foundation of solving the Boltzmann equation in this study. The algorithm combines Eulerian and Lagrangian frameworks by tracing phase-space particle trajectories backward in time, and then updates the distribution function and the self-consistent field by interpolation at stationary grid nodes.

The Boltzmann equation satisfied by the electron in graphene is:

$\frac{\partial f}{\partial t}+v_{F}\frac{k_{x}}{\left|k\right|}\frac{\partial f}{\partial x}-e(E_{0}+E_{x})\frac{\partial f}{\partial k_{x}}=S_{e-ph}$, (11)

Here, we consider the external static electric field *E*_0_ and the longitudinal electric field associated with plasmon *E_x_*, which can be calculated by the theoretical derivation (Eq. (M5) in the paper)

$E_{x}=\frac{ie(n(x,t)-n_{0})}{\varepsilon_{0}(\varepsilon_{1}\coth(\gamma_{1}d_{1})+\varepsilon_{2})}$. (12)

In the Boltzmann framework, the electron concentration *n* is related to the electron distribution function as:

$n(x,t)=\frac{g}{(2\pi)^{2}}\int f(x,k,t)dk_{x}dk_{y}$, (13)

where *g* = 4 represents degeneracy in graphene. According to Ref. [S3], the detailed procedure to simulate Eq. (11) during one time step (from *t*_n_ to *t*_n+1_) is as follows:

1. Perform a half-time step shift along the *x*-axis:

$f^{(1)}=f^{n}(x-\frac{v(k_{x},k_{y})\Delta t}{2},k_{x},k_{y})$.

Here, *v*(*k_x_*,*k_y_*) = *v*_F_ cos*θ* (*θ* = arctan(*k_y_*/*k_x_*)).

1. Compute the electric field at time *t*_n+1/2_ by computing the perturbation of electron concentration according to Eqs. (12) and (13):

$E_{x}(x,t_{n+1/2})=\frac{ige}{(2\pi)^{2}\varepsilon_{0}(\varepsilon_{1}\coth(\gamma_{1}d_{1})+\varepsilon_{2})}\int f^{(1)}dk_{x}dk_{y}$.

1. Perform a shift along the *k_x_*-axis:

.

1. Perform a second half-time step shift along the *x*-axis:

$f^{(3)}=f^{(2)}(x-\frac{v(k_{x},k_{y})\Delta t}{2},k_{x},k_{y})$.

1. Use the relaxation time approximation to deal with the collision term on the right-hand side of Eq. (11):

$f^{n+1}=f^{(3)}-(f^{(3)}-f_{0})\Delta t\Gamma(k_{x},k_{y})$,

where *Г*(*k_x_*,*k_y_*) is the scattering rate.

The advantage of the semi-Lagrangian scheme lies in its lower requirement for time step accuracy compared with explicit Eulerian ones, which circumvents the Courant-Friedrichs-Lewy (CFL) condition. The price to pay is to reconstruct the data on the grid using interpolation. Specifically, in procedures 1, 3, and 4, we use third-order spline interpolations to reconstruct the grid data after each advancement of the distribution function along the *x-* or *k_x_*-axis.

**3 Effects of Different Optical Phonon Modes in Graphene**

Three distinct optical phonon modes are critical to inelastic electron scattering in graphene [S4]. The first two, longitudinal (LO) and transverse (TO) optical phonons, have wave vectors near the **Γ** point and share an energy of *ħω*_1_=*ħω*_2_=196 meV. Also significant are zone boundary phonons near the **K** point (*ħω*_3_=160 meV), which drive intervalley processes. In order to clearly understand the influence of different modes on the plasmon instability described in the manuscript, we can characterize the influence of a particular mode by the increment of the plasmon when that mode is present alone. Since the energies of the LO and TO modes are the same, under the relaxation time approximation, these two modes have the same scattering rate with electrons. Therefore, we only need to calculate either of these two modes.

Figure S2a indicates that zone boundary phonons near the **K** point dominate the amplification of the plasmon, while the LO/TO modes have a little impact. According to Eq. (M23) in the manuscript, which describes the scattering rate between optical phonons and electrons, the scattering rates of different modes mainly depend on two factors: the frequency and the optical deformation potential of each mode. We can observe that the differences in frequencies of the three modes are not huge, but the optical deformation potential of zone boundary phonons near the **K** point is significantly greater than that of the other two modes. This results in the scattering rate of this mode being higher than that of the other two modes when the electron energy is constant, as shown in Figure S2b. Therefore, zone boundary phonons near the **K** point play a dominant role in the plasmon amplification process.


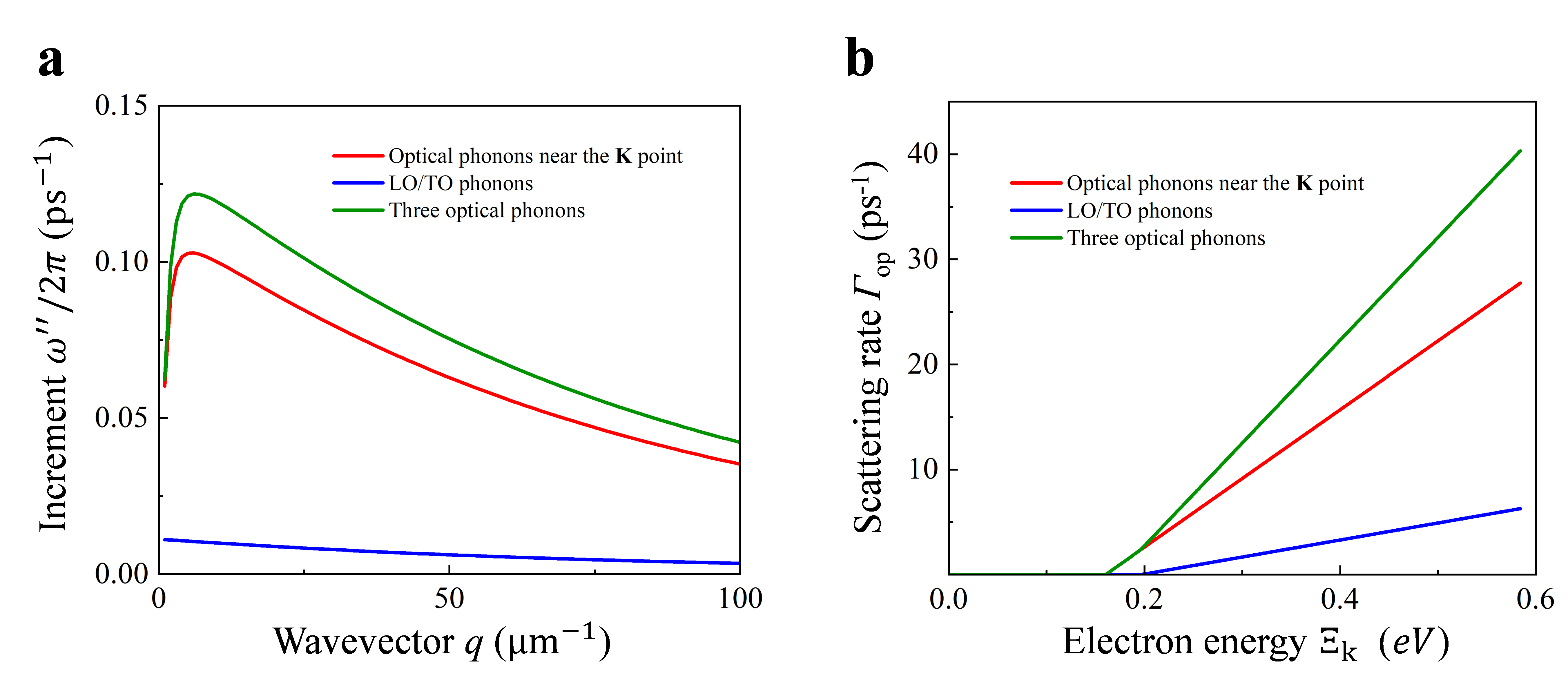


**Figure S2. The influence of different optical phonon modes on the plasmon instability. (a)** The contributions of each optical phonon mode to the increment of plasmon instability. **(b)** The scattering rates of each mode with the electrons.

**4 The saturated plasmon electric field of the graphene system**

According to Eq. (12), we can clearly observe that the plasmon electric field is directly related to the oscillating electron concentration, while the dielectric constants and the distance are fixed once the device is fabricated. Therefore, the saturation of the plasmon electric field indicates the saturation of the oscillating electron concentration. We have conducted additional simulations to study the saturated values of the oscillating electric field and electron concentration under various equilibrium electron concentrations and electron drift velocities.

Figures S3(a) and S3(b) show that, within the parameter region where instability occurs, an increase in electron concentration and electron drift velocity leads to a higher amplitude of oscillating electron concentration, thereby enhancing the electric field strength after saturation. Additionally, we find that once the system reaches saturation, the electron concentration perturbation increases to be comparable to the equilibrium concentration, i.e., 0.1 < *n*_1max_/*n*_0_ < 1. It might be a notable feature that emerges after saturation.


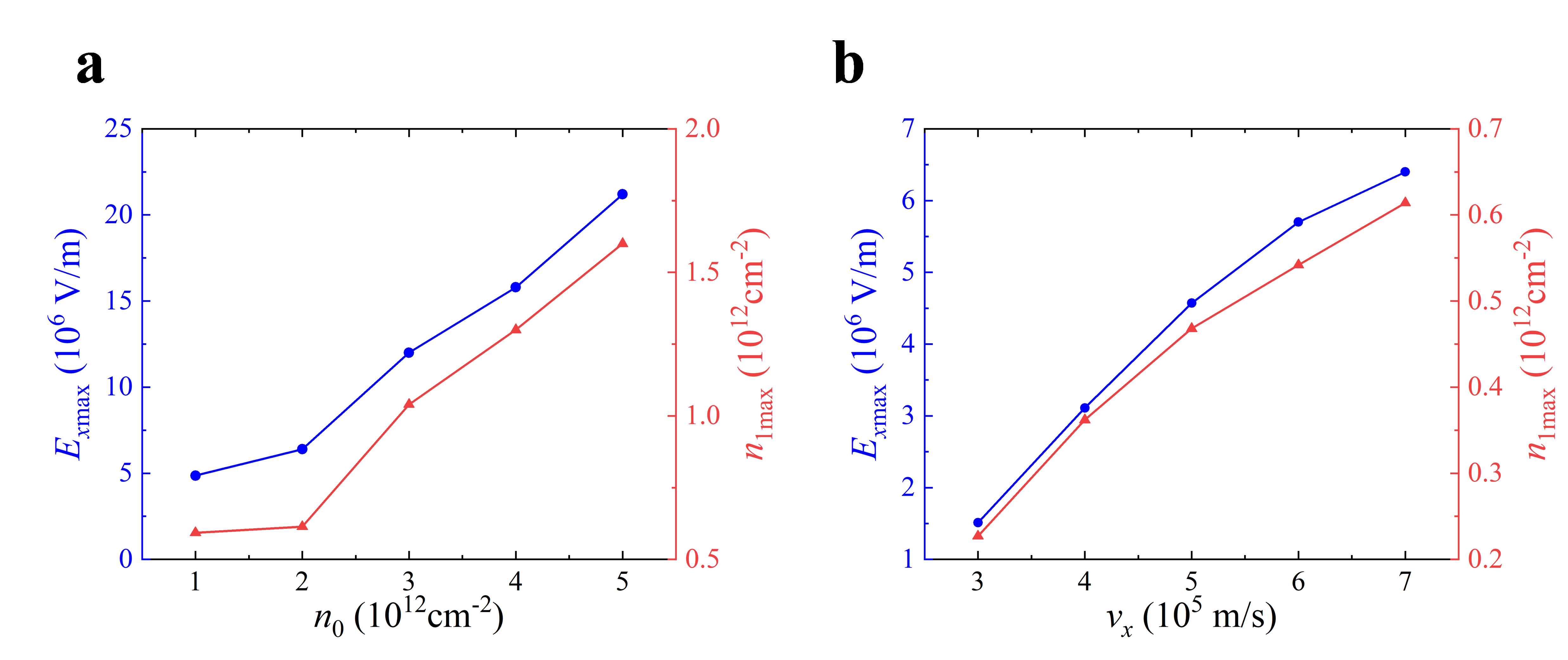


**Figure S3. The saturated plasmon electric fields and the corresponding electron concentration perturbations under different system parameters.** **(a)** The results at different equilibrium electron concentrations when the electron drift velocity is *v_x_* = 0.7*v*_F_. **(b)** The results for different electron drift velocities, with an electron concentration of *n*_0_ = 2×10^12^ cm^-2^. In both figures, the blue dot line represents the peak value of the saturated electric field *E_x_*_max_, and the red dot line represents the corresponding peak value of the oscillating electron concentration *n*_1max_.

[S1]. Crabb J, Cantos-Roman X, Jornet J M, et al. Hydrodynamic theory of the Dyakonov-Shur instability in graphene transistors. *Phys Rev B*. 2021; 104(15): 155440

[S2]. He X Y, Li R. Comparison of graphene-based transverse magnetic and electric surface plasmon modes. *IEEE J Sel Top Quantum Electron*. 2013; 20(1): 62-67.

[S3]. Sonnendrücker E, Roche J, Bertrand P, et al. The semi-Lagrangian method for the numerical resolution of the Vlasov equation. *J Comput Phys*. 1999; 149(2): 201-220.

[S4] Lichtenberger P, Morandi O, Schürrer F. High-field transport and optical phonon scattering in graphene. Phys Rev B. 2011; 84(4): 045406.
